# Transesophageal lung ultrasound score is associated with arterial oxygenation and clinical outcomes in mechanically ventilated critically ill patients

**DOI:** 10.1186/s40635-025-00818-9

**Published:** 2025-10-23

**Authors:** Daiyin Cao, Wenliang Song, Weining Zhu, Tao Yang, Xiaoxun Ma, Hao Yuan, Xiangdong Guan, Jianfeng Wu, Rui Shi, Xiang Si

**Affiliations:** 1 Department of Critical Care Medicine, The Sixth Affiliated Hospital of Sun Yat-Sen University, Guangzhou, 510655 China; 2 Department of Critical Care Medicine, The First Affiliated Hospital of Sun Yat-Sen University, Guangzhou, 510080 China; 3 Guangdong Clinical Research Centre for Critical Care Medicine, Guangzhou, 510080 China; 4 Medical Affairs Office, Tianhe District Maternal and Child Health Care Hospital of Guangzhou, Guangzhou, 510620 China

**Keywords:** Point-of-care, Transthoracic, Echocardiography, Pulmonary, PaO₂/FiO₂, Mortality, Perioperative, Hemodynamic, ICU

## Abstract

**Background:**

Transesophageal lung ultrasound (TELUS) has emerged as a novel modality that utilizes the esophageal acoustic window to obtain high-resolution images of posterior lung regions. However, its quantitative assessment and clinical relevance remain poorly explored. This study aimed to evaluate the feasibility and prognostic value of TELUS in critically ill patients, focusing on its association with arterial oxygenation and 28-day mortality.

**Methods:**

In this prospective observational study, TELUS was performed in 69 mechanically ventilated ICU patients. TELUS imaging was acquired at three esophageal levels corresponding to posterior apical, mid, and basal lung regions. A semi-quantitative TELUS score was derived and its correlation with clinical variables was analyzed. Univariate and multivariate logistic regression analyses were employed to identify predictors of 28-day mortality. Receiver operating characteristic (ROC) analysis was used to assess the predictive performance of TELUS for mortality and severe hypoxemia (PaO₂/FiO₂ ≤ 100).

**Results:**

Non-survivors had significantly higher TELUS scores compared to survivors (median 5 [IQR 4–6] vs. 4 [3–5], *P* = 0.001). Regional TELUS scores at the upper-aortic arch level and mid-esophageal level were elevated in non-survivors (*P* = 0.018 and *P* = 0.004, respectively). TELUS scores showed a significantly negative correlation with the PaO₂/FiO₂ ratio (*r* = −0.51, *P* < 0.0001), and positive correlations with PEEP (*r* = 0.32, *P* = 0.007) and SOFA scores (*r* = 0.26, *P* = 0.032). Multivariate analysis identified both SOFA (OR 1.31, 95% CI 1.08–1.63, *P* = 0.009) and TELUS scores (OR 1.72, 95% CI 1.08–2.96, *P* = 0.030) as independent predictors of 28-day mortality. ROC analysis showed that a TELUS score ≥ 4 predicted 28-day mortality and severe hypoxemia (PaO₂/FiO₂ ≤ 100), yielding areas under the ROC (AUCs) of 0.74 and 0.72, with high sensitivity (89% and 100%, respectively) and negative predictive values (92% and 100%, respectively.)

**Conclusion:**

TELUS is a feasible novel technique that provides a reliable assessment of posterior lung aeration in critically ill patients. TELUS scoring correlates with impaired oxygenation and is independently associated with 28-day mortality. These findings highlight the prognostic value of TELUS and support its potential integration into transesophageal cardiopulmonary ultrasound protocols.

**Supplementary Information:**

The online version contains supplementary material available at 10.1186/s40635-025-00818-9.

## Background

Lung ultrasound (LUS) has emerged as a pivotal bedside imaging modality in the perioperative setting and in the management of acute respiratory failure in critically ill patients [1–3]. International consensus guidelines endorse the use of transthoracic lung ultrasound (TTLUS) for the rapid detection of pulmonary abnormalities, such as consolidation, pleural effusion, and pneumothorax [4–6], with diagnostic accuracy comparable to that of chest computed tomography (CT) [7, 8]. Furthermore, TTLUS scoring offers a semi-quantitative assessment of pulmonary involvement and has been utilized to monitor lung re-aeration following therapeutic interventions [9–11].

However, in clinical practice, patient-related factors such as obesity, massive edema, heavy musculature, subcutaneous emphysema, surgical dressings, or thoracic trauma can impair the acquisition of adequate TTLUS images [12]. These limitations are further compounded by the supine positioning commonly required in ICU settings, which hampers the assessment of gravity-dependent lung regions—dorsal areas frequently involved in pathologies, such as pneumonia and acute respiratory distress syndrome (ARDS). Moreover, scapular interference precludes visualization of the posterior upper lung zones, rendering comprehensive transthoracic assessment challenging [12, 13].

Transesophageal lung ultrasound (TELUS) has recently been proposed as a novel technique that leverages the esophageal acoustic window to obtain high-resolution images of posterior lung regions adjacent to the mediastinum [14, 15]. This approach facilitates visualization of dorsal lung areas that are often challenging to assess via transthoracic methods, particularly the blind spots created by the scapulae. Several studies have reported that TELUS can detect lung consolidations [16, 17] and pleural effusions [18, 19] with high diagnostic sensitivity. Given these advantages, and the feasibility of acquiring TELUS views during routine transesophageal echocardiography (TEE) examination, TELUS has been advocated to be integrated into the standard TEE protocols in perioperative and critical care settings, providing complementary pulmonary information alongside hemodynamic assessment [20].

Despite its promising potential, TELUS remains in the early stages of investigation, with limited data on quantitative scoring systems and its clinical utility yet to be fully established [21]. The present study aims to assess the feasibility and prognostic value of TELUS in critically ill patients, focusing on its association with arterial oxygenation and 28-day mortality.

## Methods

### Study population

This prospective, observational, cohort study was conducted between April 2019 and October 2020 in an 18-bed ICU at the First Affiliated Hospital of Sun Yat-sen University, Guangzhou, China. Patients were eligible for inclusion if they met all the following criteria: (i) age ≥ 18 years; (ii) ICU stay > 24 h; (iii) mechanically ventilated; and (iv) underwent TEE examination due to respiratory or circulatory failure. Respiratory failure was defined as a PaO₂ < 60 mmHg with or without PaCO₂ > 50 mmHg, requiring invasive mechanical ventilation. Circulatory failure was defined as persistent hypotension necessitating vasopressor support to maintain systolic blood pressure > 90 mmHg or mean arterial pressure > 65 mmHg. Exclusion criteria included: (i) pregnancy or peripartum status; (ii) contraindications to TEE, such as unrepaired tracheoesophageal fistula, previous esophageal surgery, esophageal obstruction or stricture, esophageal varices or diverticula, gastrointestinal or esophageal bleeding, oropharyngeal pathology, and severe coagulopathy; (iii) unsuccessful TEE probe placement or inadequate TELUS image acquisition; and (iv) inability to obtain informed consent from the patient or their legal representative. The study protocol was approved by the local ethics committee (2015–120) and registered with the China Clinical Trial Registry (ChiCTR: 1900022172). Informed consent was obtained from each patient or from the patient’s legally authorized representative if the patient was unable to provide consent.

### TELUS examination protocol

**Fig. 1:**
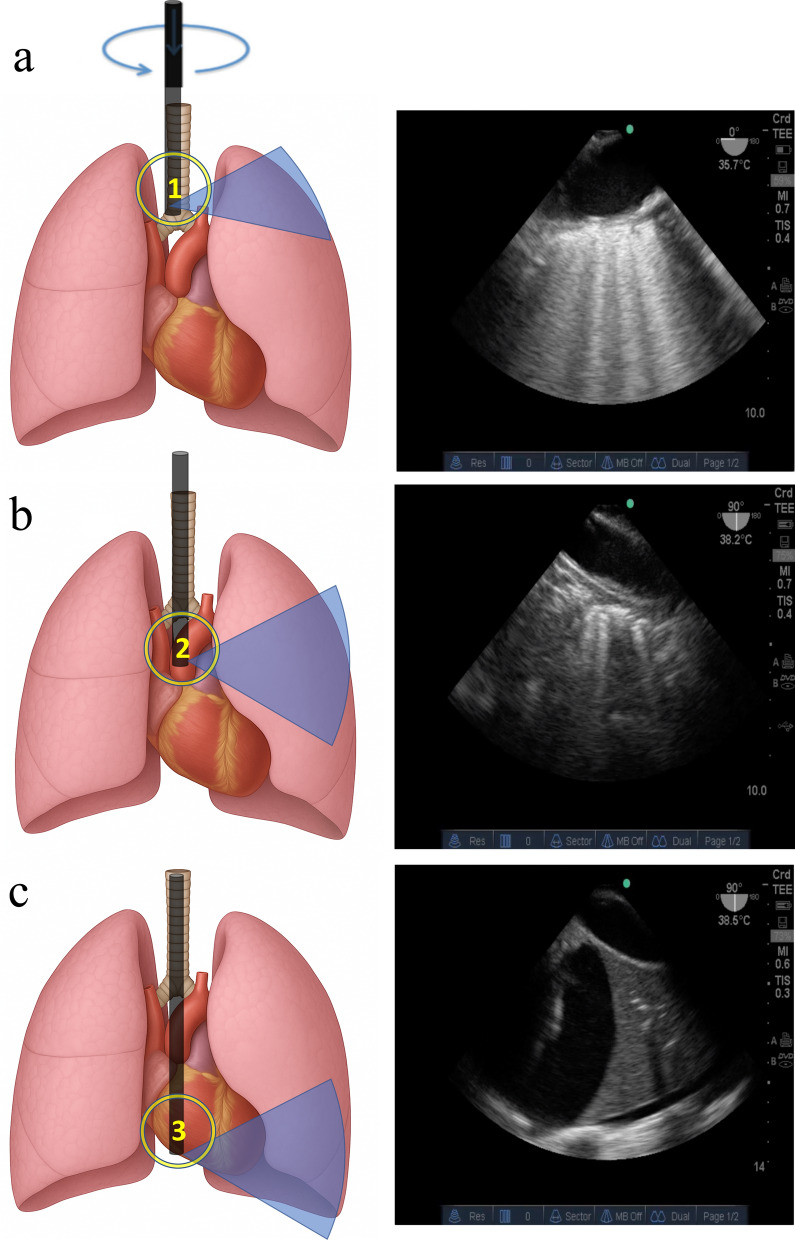
Schematic illustration of TELUS-scanned lung regions with corresponding ultrasound images, referenced to the aortic arch and descending thoracic aorta. **a** Apical-posterior lung region, corresponding to the aortic arch at the level of the origin of the left subclavian artery. **b** Mid-posterior lung region, corresponding to the descending thoracic aorta at the level of the left superior pulmonary vein. **c** Basal posterior lung region, corresponding to the descending thoracic aorta at the level of the origin of the inferior vena cava. Electronic multiplane angle is displayed in the upper-right corner: 0° corresponds to the long axis of the aortic arch (panel a), and 90° corresponds to the long axis of the descending thoracic aorta (panels b and c). TELUS transesophageal lung ultrasound

All enrolled patients underwent TELUS within 24 h of ICU admission using either an M-Turbo system (FUJIFILM Sonosite, Bothell, WA, USA) or GE Venue system (GE Healthcare, Madison, WI, USA). A multiplane 5-MHz transesophageal probe was used to acquire lung images. Sedation with intravenous midazolam or propofol was administered prior to probe insertion to ensure patient comfort and procedural tolerance. The probe was introduced blindly by advancing the tip into the posterior pharynx in the midline position, allowing passive flexion of the transducer to facilitate esophageal entry. TELUS was performed in conjunction with the aortic arch and descending thoracic aorta. Three lung regions were identified as previously described, namely, the posterior apical, mid, and basal lung zones [14]. The schematic illustration of the scanned areas with corresponding TELUS images is shown in Fig. 1.

The TELUS examination commenced with the acquisition of the mid-esophageal four-chamber view. Using the left superior pulmonary vein as an anatomical landmark, clockwise rotation of the probe from this position enabled visualization of the superoposterior lung fields. At a multiplane angle of 0°, the short axis of the descending aorta was acquired; then electronic rotation to 90° permitted scanning of the lungs in the longitudinal axis. Subsequently, gradual withdrawal of the probe to the level of the origin of the left subclavian artery allowed assessment of the apical-posterior lung region. At a multiplane angle of 90°, the short axis of the aortic arch was obtained, followed by 0° rotation for longitudinal scanning. Finally, advancing the probe distally to the level of the inferior vena cava origin facilitated imaging of the inferoposterior lung zones, again using 0° for short axis and 90° for longitudinal views. In each lung area, both short- and long-axis views were acquired by adjusting the transducer angle from 0° to 90° (Supplemental Video) [14]. The TELUS examination was performed by one trained clinician with advanced competence in critical care echocardiography (X.S.). The TELUS images or videos were stored for analysis. Based on both institutional experience and previous literature [20], imaging of the right hemithorax is technically more challenging due to interference from cardiac and vertebral structures, which often obscure visualization of the posterior right lung. Therefore, the right hemithorax was excluded from the present analysis.

### TELUS scores

TELUS images and videos were reviewed offline by an expert intensivist (D.C.), who was blinded to patient identity. To assess inter-observer reproducibility, all videos were independently re-evaluated by a second blinded investigator (W.S.). Each region was scored according to standardized LUS patterns as follows [4]: a score of 0 was assigned when lung sliding was present with A-lines or no more than two isolated B-lines; a score of 1 was given for the presence of multiple well-defined B-lines; a score of 2 indicated multiple coalescent B-lines; and a score of 3 was used when lung consolidation (i.e., “tissue-like” areas with or without air bronchograms) or pleural effusion was observed. The TELUS score was assigned based on the most severe lung pattern at end-expiration. The highest score identified in each region was recorded, and the total TELUS score was calculated, with a maximum score of nine.

### Data collection

Baseline demographics (age, sex, diagnosis, comorbidities, etc.) were collected at enrollment. At the time of TELUS examination, arterial oxygenation (PaO₂ and PaO₂/FiO₂ ratio), blood lactate levels, and mechanical ventilator parameters were recorded. Organ support including vasopressor use, mechanical ventilation, and continuous renal replacement therapy (CRRT) was also documented. Severity of illness was assessed within 24 h of ICU admission using the Sequential Organ Failure Assessment (SOFA) score. Clinical outcomes included ICU length of stay, total hospital length of stay, and 28-day mortality.

### Statistical analysis

The normality of data distribution was assessed using the Kolmogorov–Smirnov test. Normally distributed continuous variables were presented as mean ± standard deviation (SD), while non-normally distributed data were reported as medians (interquartile range). Categorical variables were expressed as counts (*n*) and percentages (%). Inter-observer reproducibility was quantified using the intraclass correlation coefficient (ICC) for continuous variables. Comparisons of variables in survivors and non-survivors were analyzed using either an unpaired Student’s *t* test or a Mann–Whitney *U* test, depending on data distribution. Comparisons of proportions were assessed by either the chi-squared test or Fisher’s exact test.

Correlations between TELUS scores and clinical parameters were analyzed using Spearman correlation coefficients and presented as a correlation matrix. Univariate logistic regression was employed to identify factors associated with 28-day mortality; variables with *P* < 0.20 were included in a multivariate logistic regression model. This approach, consistent with previous statistical [22–24] and clinical studies [25, 26], aimed to avoid excluding potentially relevant predictors that might become significant after adjustment. Odds ratios (ORs) and 95% confidence intervals (CIs) were calculated to determine independent predictors of mortality.

Receiver operating characteristic (ROC) curve analysis was used to evaluate the predictive performance of TELUS scores for impaired oxygenation and 28-day mortality. The area under the ROC curve (AUC) was calculated, and optimal cutoff values were determined using the Youden index, with sensitivity, specificity, positive predictive values (PPVs) and negative predictive values (NPVs) reported accordingly.

All statistical analyses were performed using GraphPad Prism (version 9.5.1; GraphPad Software, San Diego, CA, USA) and R (version 4.3.1; R Studio, version 1.0.136). A two-tailed *p* value < 0.05 was considered statistically significant.

## Results

### Patient characteristics

A total of 78 critically ill patients underwent transesophageal ultrasound examination, of whom 9 were excluded due to inadequate TELUS imaging (Figure S1), resulting in 69 patients included in the final analysis. Of these, 51 survived within 28 days, yielding a 28-day mortality rate of 26.1%. Baseline characteristics are summarized in Table 1**.** Compared with survivors, non-survivors had significantly higher serum lactate levels, a greater proportion of vasopressor use, and elevated SOFA scores upon ICU admission (all *P* < 0.05). No significant differences were observed in other baseline variables between the two groups (*P* > 0.05). The inter-observer agreements between physicians for the assessment of TELUS analysis were 0.85 (95% CI 0.71–0.90).

**Table 1:** Comparison of baseline characteristics between survivors and non-survivors

Baseline characteristics	Total (*n* = 69)	Survivors (*n* = 51)	Non-survivors (*n* = 18)	*P* value^1^
Sex [male, *n* (%)]	37 (53.6%)	26 (51.0%)	11 (61.1%)	0.435
Age [median (IQR)], years	61 (49–75)	59 (49–79)	64 (47–75)	0.868
Admission diagnosis, *n* (%)				
Circulatory failure	50 (72.4%)	34 (66.7%)	16 (88.9%)	0.099
Septic shock	29 (42.0%)	19 (37.3%)	10 (55.6%)	0.106
Vasoplegic non-septic	6 (8.7%)	6 (11.8%)	0 (0.0%)	0.182
Hypovolemic shock	5 (7.2%)	4 (7.8%)	1 (5.6%)	0.999
Cardiac shock	6 (8.7%)	3 (5.9%)	3(16.7%)	0.181
Obstructive shock	4 (5.8%)	2 (3.9%)	2 (11.1%)	0.158
Respiratory failure	19 (27.5%)	17 (33.3%)	2 (11.1%)	0.099
Peumonia	9 (13.0%)	8 (15.7%)	1 (5.6%)	0.682
Post-vascular surgery	6 (8.7%)	5 (9.8%)	1 (5.6%)	0.675
Other	4 (5.8%)	4 (7.8%)	0 (0.0%)	0.570
ARDS, *n* (%)	50 (72.5%)	35(68.6%)	15(83.3%)	0.381
Comorbidities, *n* (%)				
Hypertension	24 (34.8%)	18 (35.3%)	6 (33.3%)	0.885
Diabetes mellitus	12 (17.4%)	10 (19.6%)	2 (11.1%)	0.504
Cardiovascular disease	11 (15.9%)	9 (17.6%)	2 (11.1%)	0.722
Chronic renal disease	3 (4.3%)	2 (4.0%)	1 (5.5%)	0.559
Organ support, *n* (%)				
Vasopressors	44 (63.8%)	28 (54.9%)	16 (88.9%)	0.009
CRRT	16 (23.2%)	9 (17.6%)	7 (38.9%)	0.099
Mechanical ventilation	69 (100.0%)	51 (100.0%)	18 (100.0%)	1.000
Lactate [median (IQR)], mmol/L	1.9 (1.1–3.3)	1.8 (1.1–2.8)	2.9 (1.3–6.3)	0.031
SOFA score [median (IQR)]	8 (6–12)	7 (5– 9)	12 (8–14.5)	< 0.001
ICU length of stay [median (IQR)], days	7 (4–13)	8 (4–13)	6 (3–12)	0.216
Hospital length of stay [median (IQR)], days	19 (11.5–35)	26 (15–47)	8 (4–19)	< 0.001

^1^Pearson’s chi-squared test; Fisher’s exact test; Wilcoxon rank sum test; Wilcoxon rank sum exact test

Survivors vs. non-survivors refer to 28-day mortality. *CRRT* continuous renal replacement therapy, *SOFA* sequential organ failure assessment, *ICU* intensive care unit

### Comparison of TELUS scores and respiratory parameters

Non-survivors exhibited significantly higher total TELUS scores compared to survivors (5[4–6] vs. 4[3–5], *P* = 0.001) (Table 2). Specifically, TELUS scores at the upper esophageal aortic arch level (0.5[0–2] vs. 0[0–1], *P* = 0.018) and mid-esophageal descending aorta level (2[1–2.3] vs. 1[0–2], *P* = 0.004) were both significantly elevated in the non-survivor group. In contrast, no significant difference was observed at the lower esophageal descending aorta level (3[2, 3] vs. 3[2, 3], *P* = 0.594). The PaO₂/FiO₂ ratio tended to be lower in non-survivors but did not reach statistical significance (169[129–247] mmHg vs. 235[131–364] mmHg, *P* = 0.088). No significant differences were found in ventilator parameters, including PEEP, pressure support, and tidal volume (all *P* > 0.05).

**Table 2:** Comparison of TELUS scores, oxygenation, and ventilator settings between survivors and non-survivors

Variable	Total (*n* = 69)	Survivors (*n* = 51)	Non-survivors (*n* = 18)	*P* value^1^
Total TELUS score	4 (3–5)	4 (3–5)	5 (4–6)	0.001
Upper esophageal (aortic arch) TELUS score	0 (0–1)	0 (0–1)	0.5 (0–2)	0.018
Mid-esophageal (descending aorta) TELUS score	1 (0–2)	1 (0–2)	2 (1–2.3)	0.004
Lower esophageal (descending aorta) TELUS score	3 (2–3)	3 (2–3)	3 (2–3)	0.594
PaO₂/FiO₂ ratio	218 (133–325)	235 (131–364)	169 (129–247)	0.088
PaO₂, mmHg	98 (75–142)	103 (71–150)	93 (80–126)	0.496
Ventilator parameters				
Positive end-expiratory pressure (PEEP, cmH₂O)	6 (5–8)	5 (5–8)	6.5 (5–9.3)	0.444
Pressure support (PS, cmH₂O)	12 (12–13)	12 (12–13)	12.5 (12–14.3)	0.116
Tidal volume (TV, mL/kg PBW)	7.3 (6.2–8.2)	7.3 (6.4–8.2)	7.2 (5.6–8.1)	0.311

^1^Wilcoxon rank sum test; Wilcoxon rank sum exact test

Survivors vs. non-survivors refer to 28-day mortality. *ICU* intensive care unit, *TELUS* transesophageal lung ultrasound, *PaO*_*2*_*/FiO*_*2*_ oxygen arterial partial pressure over inspired fraction of oxygen, *PEEP* positive end-expiratory pressure, *PS* pressure support, *PBW* predicted body weight, *TV* tidal volume

### Correlation analysis of TELUS scores

**Fig. 2:**
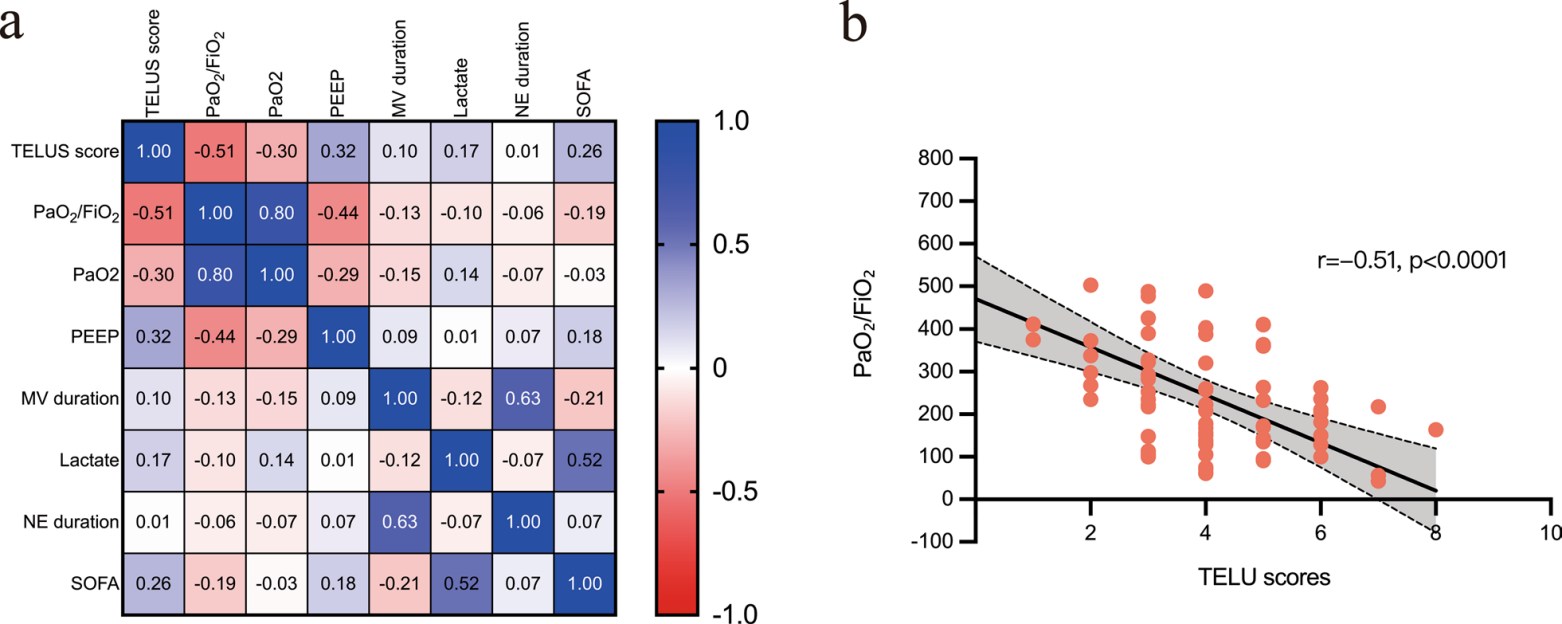
Associations between TELUS scores and clinical variables. **a** Correlation matrix illustrating the relationships between TELUS scores and relevant clinical parameters. **b** Scatter plot showing the negative correlation between TELUS scores and the PaO₂/FiO₂ ratio. *TELUS* transesophageal lung ultrasound, *PaO₂/FiO₂* oxygen arterial partial pressure over inspired fraction of oxygen, *PEEP* positive end-expiratory pressure, *MV* mechanical ventilation, *NE* norepinephrine, *SOFA* sequential organ failure assessment

A correlation matrix was constructed to explore the associations between TELUS scores and clinical variables (Fig. 2a). TELUS scores showed a significant negative correlation with both the PaO₂/FiO₂ ratio (*r* = −0.51, *P* < 0.0001) (Fig. 2b) and PaO₂ (*r* = −0.30, *P* = 0.012), and a positive correlation with PEEP levels (*r* = 0.32, *P* = 0.007) and SOFA scores (*r* = 0.26, *P* = 0.032). Notably, the strongest association was observed between TELUS scores and the PaO₂/FiO₂ ratio, exceeding the correlations with other parameters such as PEEP (–0.51 vs. 0.32, *P* < 0.001), suggesting that higher TELUS scores are closely associated with more severely impaired oxygenation.

### Multivariate analysis and predictive value of TELUS scores

**Fig. 3:**
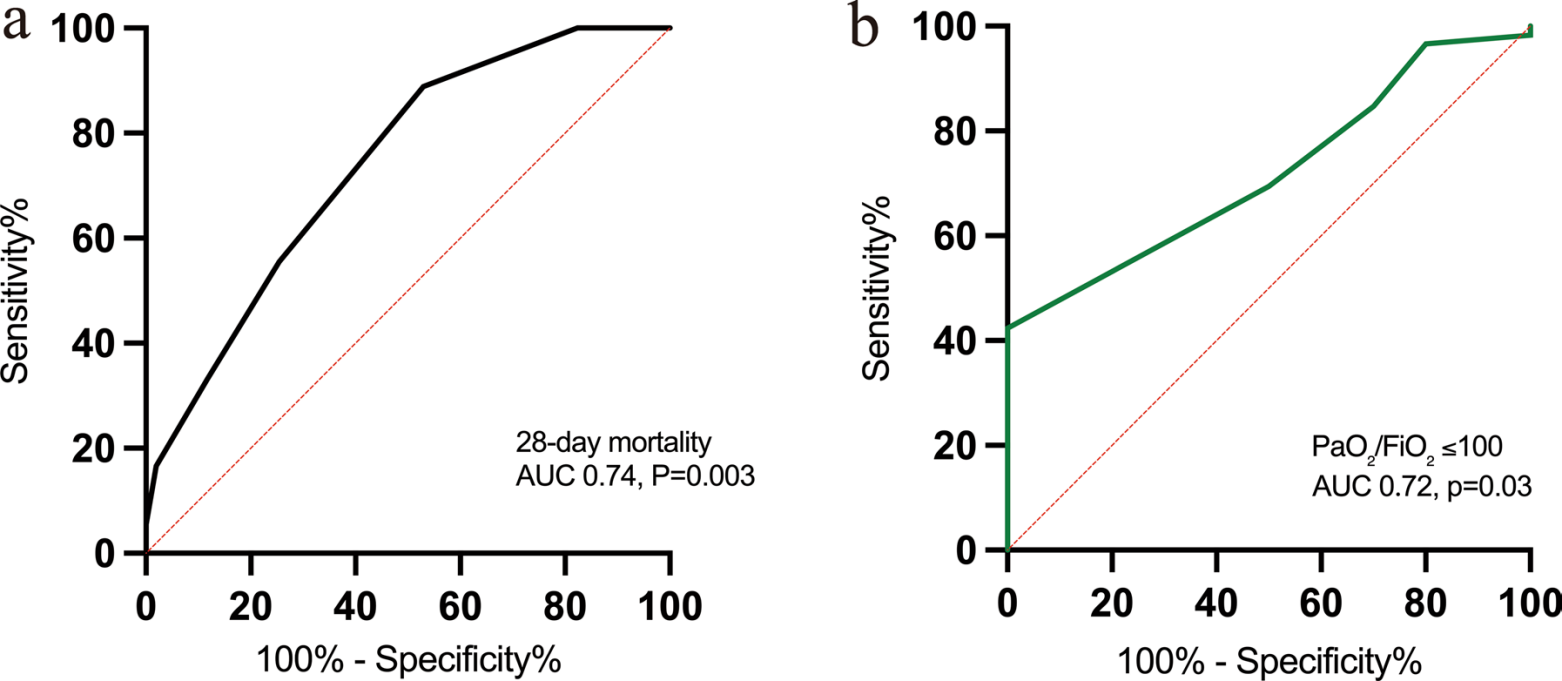
ROC curves of the TELUS score for predicting the **a** 28-day mortality and **b** severe hypoxemia (PaO₂/FiO₂ ratio ≤ 100) in critically ill patients. *AUC* area under the receiver operating characteristic curve, *ROC* receiver operating characteristic, *TELUS* transesophageal lung ultrasound, *PaO₂/FiO₂* oxygen arterial partial pressure over inspired fraction of oxygen

Univariate analysis determined SOFA score (*P* < 0.0001), TELUS score (*P* = 0.003), lactate level (*P* = 0.064), and central venous pressure (CVP, *P* = 0.138) as potential predictors of 28-day mortality (threshold *P* < 0.2), and these variables were subsequently included in the multivariable logistic regression model. Multivariable analysis revealed that SOFA score (OR: 1.31, 95% CI 1.08–1.63, *P* = 0.009) and TELUS score (OR: 1.72, 95% CI 1.08–2.96, *P* = 0.030) were independent predictors of 28-day mortality (Table 3). ROC curve analysis further indicated that a TELUS score ≥ 4 yielded an AUC of 0.74 (95% CI 0.62–0.87) for discriminating survivors from non-survivors (Fig. 3a), and an AUC of 0.72 (95% CI 0.58–0.86) for identifying patients with PaO₂/FiO₂ ≤ 100 (Fig. 3b). TELUS exhibited high sensitivity for predicting 28-day mortality and severe hypoxemia (89% and 100%, respectively) and high NPVs (92% and 100%, respectively). However, its specificity (47% and 42%) and PPVs (37% and 23%) remained limited (Table 4).

**Table 3:** Univariate and multivariate logistic regression analyses of TELUS score for predicting 28-day mortality

Variable	Univariate analysis^1^			Multivariate analysis		
	OR	95% CI	*P* value	OR	95% CI	*P* value
Age	1.00	0.97–1.04	0.862	–	–	–
Lactate	1.15	0.99–1.35	0.064	0.95	0.76–1.16	0.641
SOFA score	1.33	1.14–1.60	< 0.0001	1.31	1.08–1.63	0.009
TELUS score	1.93	1.28–3.11	0.003	1.72	1.08–2.96	0.030
PEEP	1.16	0.90–1.49	0.243	–	–	–
CVP	1.12	0.96–1.30	0.138	1.10	0.93–1.32	0.271

^1^Parameters with *p* value < 0.20 between groups are included in the multivariate regression analysis

*SOFA* sequential organ failure assessment, *TELUS* transesophageal lung ultrasound, *PEEP* positive end-expiratory pressure, *CVP* central venous pressure, *OR* odds ratio, *CI* confidence interval

**Table 4:** Diagnostic ability of the TELUS score to predict 28-day mortality and severe hypoxemia (PaO₂/FiO₂ ≤ 100)

Outcome	AUC	95% CI	*P* value	Threshold	Sensitivity (%)	Specificity (%)	PPV (%)	NVP (%)
28-day mortality	0.740	0.615–0.865	0.003	≥ 4	89 (67–98)	47 (34–60)	37(24–53)	92(74–99)
PaO₂/FiO₂ ≤ 100	0.720	0.579–0.860	0.030	≥ 4	100 (72–100)	42 (31–55)	23(11–38)	100(86–100)

*AUC* area under the receiver operating characteristic curve, *PPV* positive predictive value, *NPV* negative predictive value, *ICU* intensive care unit, *TELUS* transesophageal lung ultrasound, *PaO*_*2*_*/FiO*_*2*_ oxygen arterial partial pressure over inspired fraction of oxygen *CI* confidence interval

## Discussion

The present study evaluated the clinical utility of TELUS in critically ill patients, with a focus on its association with oxygenation and 28-day mortality. Our findings showed a significant negative correlation between TELUS scores and PaO₂/FiO₂ ratios, with non-survivors exhibiting significantly higher TELUS scores compared to survivors. Furthermore, both univariate and multivariate logistic regression analyses indicated TELUS scores as independent predictors of 28-day mortality. ROC curve analysis revealed that a TELUS score of ≥ 4 yielded high sensitivity and NPV for identifying patients at risk of severe hypoxemia and increased 28-day mortality.

LUS has become a widely accepted noninvasive, repeatable, and bedside tool in the ICU for evaluating pulmonary pathologies [1–3] and it has been incorporated into critical care ultrasound guidelines [4–6]. The TTLUS score, calculated as the sum of individual lung regional scores, has been validated as a semi-quantitative method for assessing aeration loss due to various lung conditions, such as consolidation, pneumothorax, and pulmonary edema [4]. This score reflects the severity of lung injury [27] and is associated with worse patient outcomes [28]. It has been applied to monitor the adverse effects of fluid resuscitation [11], assess lung recruitment [9], and track re-aeration in response to antibiotic therapy [10]. In addition, a previous study reported that the extent of lung consolidation correlated with both impaired oxygenation and increased mortality in critically ill patients [29].

However, the application of TTLUS may be constrained by chest wall factors, such as obesity, muscularity, subcutaneous emphysema, wound dressings, trauma, or patient positioning, all of which can impair image acquisition [12]. These challenges are particularly pronounced in supine patients, in whom dependent lung regions, which are common sites for consolidation, are often inadequately visualized. In addition, the presence of the scapulae can obscure the posterior upper lung zones, making them blind spots for TTLUS [13].

TELUS has been shown to be highly sensitive for detecting pleural effusions adjacent to the descending aorta, with a reported sensitivity of 97% and specificity of 100% when compared to CT as the reference standard [18]. In addition to its diagnostic accuracy, TELUS also provides a reliable estimation of pleural fluid volume [19, 30]. Furthermore, increased density detected by TELUS in the dependent regions of the left lung has shown good correlation with CT-assessed consolidation and PaO₂/FiO₂ ratios [16]. Nevertheless, these studies generally focused on a single plane of the descending aorta only. In 2016, Cavayas et al. proposed a systematic transesophageal approach to lung ultrasound, introducing three TELUS planes: at the level of the aortic arch, the mid-descending aorta, and the lower descending aorta [14]. This technique enables the simultaneous acquisition of pulmonary data during a TEE examination by overcoming chest wall limitations, obviating the need for patient repositioning, and facilitating integrated cardiopulmonary assessment via the same probe [15]. Weber et al. further advocated for the routine incorporation of TELUS as an extension of TEE to enhance comprehensive cardiopulmonary evaluation [20]. However, a validated scoring system for TELUS is still lacking, and its diagnostic accuracy has not yet been rigorously assessed.

To our knowledge, this is the first prospective study to evaluate a TELUS-based scoring approach in critically ill patients. Our results showed that TELUS scores were significantly associated with both impaired arterial oxygenation and the severity of multi-organ dysfunction. Notably, the high sensitivity and NPVs of TELUS scoring underscore its potential as a reliable screening tool for “ruling-out” severe hypoxemia and adverse outcomes. Specifically, a low TELUS score can help identify patients at lower risk of 28-day mortality or profound oxygenation impairment. While TELUS has yet to be broadly implemented in routine clinical practice, these preliminary findings suggest its potential utility in the early risk stratification of critically ill patients.

In our cohort, TELUS scores correlated positively with PEEP, a finding that may reflect lung severity rather than PEEP being ineffective at improving posterior aeration. The concomitant negative correlation between PEEP and PaO₂/FiO₂, consistent with previous observational studies [31], further supports this interpretation. Patients with more severe lung injury were more likely to receive higher PEEP, but for the individual per se, empirically set PEEP levels (without lung recruitment monitoring) may still be insufficient to restore aeration in the dependent posterior regions, resulting in high TELUS scores and impaired oxygenation.

These findings indicate the potential value of TELUS scores in guiding personalized ventilatory strategies. By directly visualizing posterior lung aeration, TELUS can provide dynamic feedback on the effects of PEEP titration and recruitment maneuvers [32]. Moreover, when posterior aeration does not improve despite these interventions, TELUS may help guide alternative therapy, such as positioning (e.g., proning) or the management of pleural effusions. In this way, TELUS offers region-specific, real-time information that complements lung assessment.

Nevertheless, despite these promising implications, the widespread use of TELUS is limited by practical constraints (probe insertion, operator expertise, and patient tolerance), making it less feasible for routine monitoring compared with TTLUS. However, in selected patients, where transthoracic imaging is inadequate, or in those with unexplained hypoxemia or hypotension requiring TEE examination [33], TELUS could be integrated into a transesophageal cardiopulmonary ultrasound protocol to enhance diagnostic and prognostic assessment in critical care.

We acknowledge several limitations of this study. First, the study did not incorporate TEE assessment, although TELUS and TEE can be performed using the same probe. We focused on pulmonary imaging to evaluate the prognostic value of TELUS through a pragmatic lens, laying the groundwork for future studies on integrated transesophageal cardiopulmonary assessment. Second, TTLUS data were not collected concurrently. Combining TTLUS and TELUS may provide a more comprehensive evaluation of lung and warrant further investigation. Third, only the left hemithorax was assessed due to anatomical constraints. However, previous literature has also reported that the right hemithorax is more challenging to evaluate, as cardiac interposition frequently blocks an adequate view [20]. Fourth, assigning a score of 3 to tissue-like patterns, independently of its dimension, may overestimate aeration loss; nevertheless, previous studies have shown that both global and regional LUS scores correlate strongly with lung tissue density assessed by CT [8]. Future studies could refine the scoring system by incorporating the proportion of lung involved. Fifth, as an observational study, we did not assess the utility of TELUS in guiding interventions such as ventilator recruitment maneuvers. Finally, the relatively small sample size and single-center design necessitate larger, multicenter studies to validate these findings.

## Conclusion

The present study evaluated the clinical utility of TELUS in critically ill patients. Our findings indicated that TELUS scores were significantly associated with impaired arterial oxygenation and exhibited high sensitivity and NPVs in predicting 28-day mortality, supporting their potential as a reliable screening tool to rule out severe hypoxemia and adverse outcomes. Compared with conventional TTLUS, TELUS offers distinct advantages in acquiring posterior lung images, particularly in patients who are unable to tolerate positional changes or have chest wall limitations. By enabling direct visualization of posterior lung regions adjacent to the thoracic aorta, TELUS provides meaningful diagnostic value and lays the groundwork for further development of integrated transesophageal cardiopulmonary ultrasound protocols in perioperative or critical care settings.

## Supplementary Information

Supplementary material 1.

## Data Availability

The data sets used in the present study are available from the corresponding author on reasonable request.

## Abbreviations

AUROC: Area under the receiver operating characteristic curve
CI: Confidence interval
CRRT: Continuous renal replacement therapy
CT: Computed tomography
CVP: Central venous pressure
ICC: Intraclass correlation coefficient
LUS: Lung ultrasound
NPV: Negative predictive value
OR: Odds ratio
PEEP: Positive end-expiratory pressure
PPV: Positive predictive value
ROC: Receiver operating characteristic
SD: Standard deviation
SOFA: Sequential organ failure assessment
TEE: Transesophageal echocardiography
TELUS: Transesophageal lung ultrasound
TTLUS: Transthoracic lung ultrasound
