# Supplementary material for: Transesophageal lung ultrasound score is associated with arterial oxygenation and clinical outcomes in mechanically ventilated critically ill patients
Source: Intensive Care Med Exp. 2025 Oct 23;13:107. doi: 10.1186/s40635-025-00818-9 (PMC12549491; doi:10.1186/s40635-025-00818-9)
Supplement: Supplementary file 1 — Supplementary material 1. [file 40635_2025_818_MOESM1_ESM.pptx]

## Slide 1
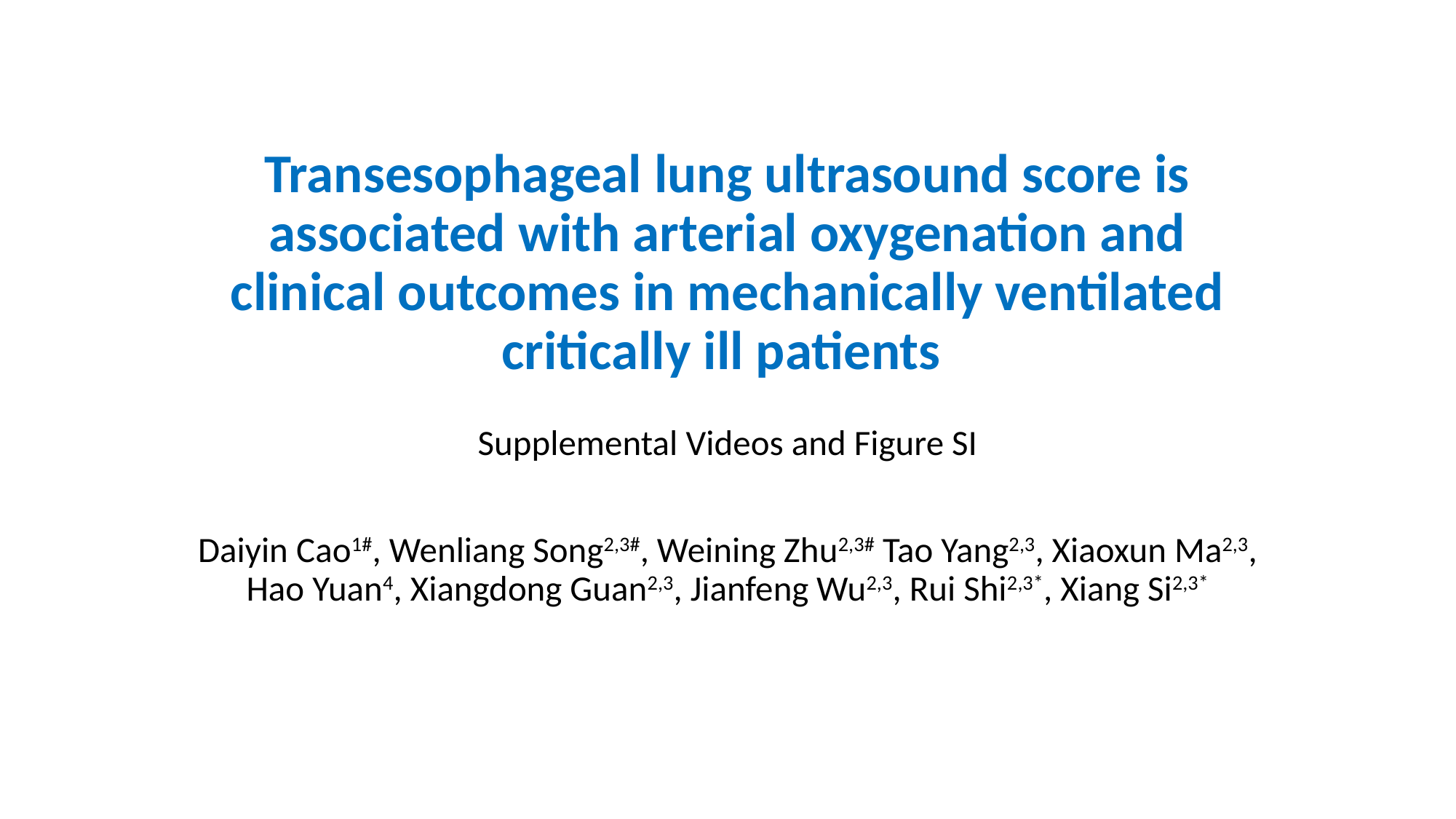

# Transesophageal lung ultrasound score is associated with arterial oxygenation and clinical outcomes in mechanically ventilated critically ill patients
Supplemental Videos and Figure SI
Daiyin Cao1#, Wenliang Song2,3#, Weining Zhu2,3# Tao Yang2,3, Xiaoxun Ma2,3, Hao Yuan4, Xiangdong Guan2,3, Jianfeng Wu2,3, Rui Shi2,3*, Xiang Si2,3*

## Slide 2
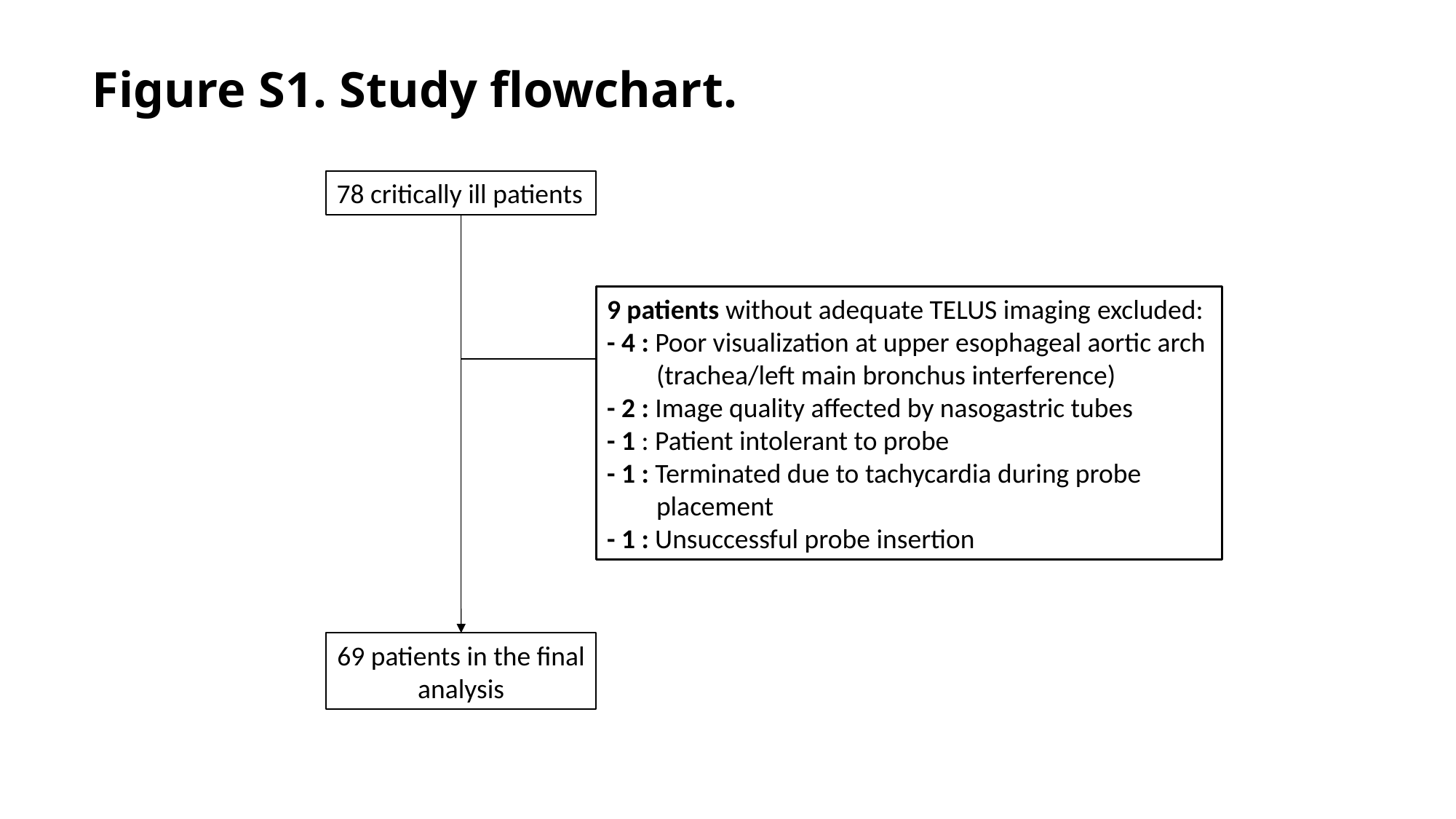

# Figure S1. Study flowchart.
78 critically ill patients
9 patients without adequate TELUS imaging excluded:
- 4 : Poor visualization at upper esophageal aortic arch
 (trachea/left main bronchus interference)
- 2 : Image quality affected by nasogastric tubes
- 1 : Patient intolerant to probe
- 1 : Terminated due to tachycardia during probe
 placement
- 1 : Unsuccessful probe insertion
69 patients in the final analysis

## Slide 3
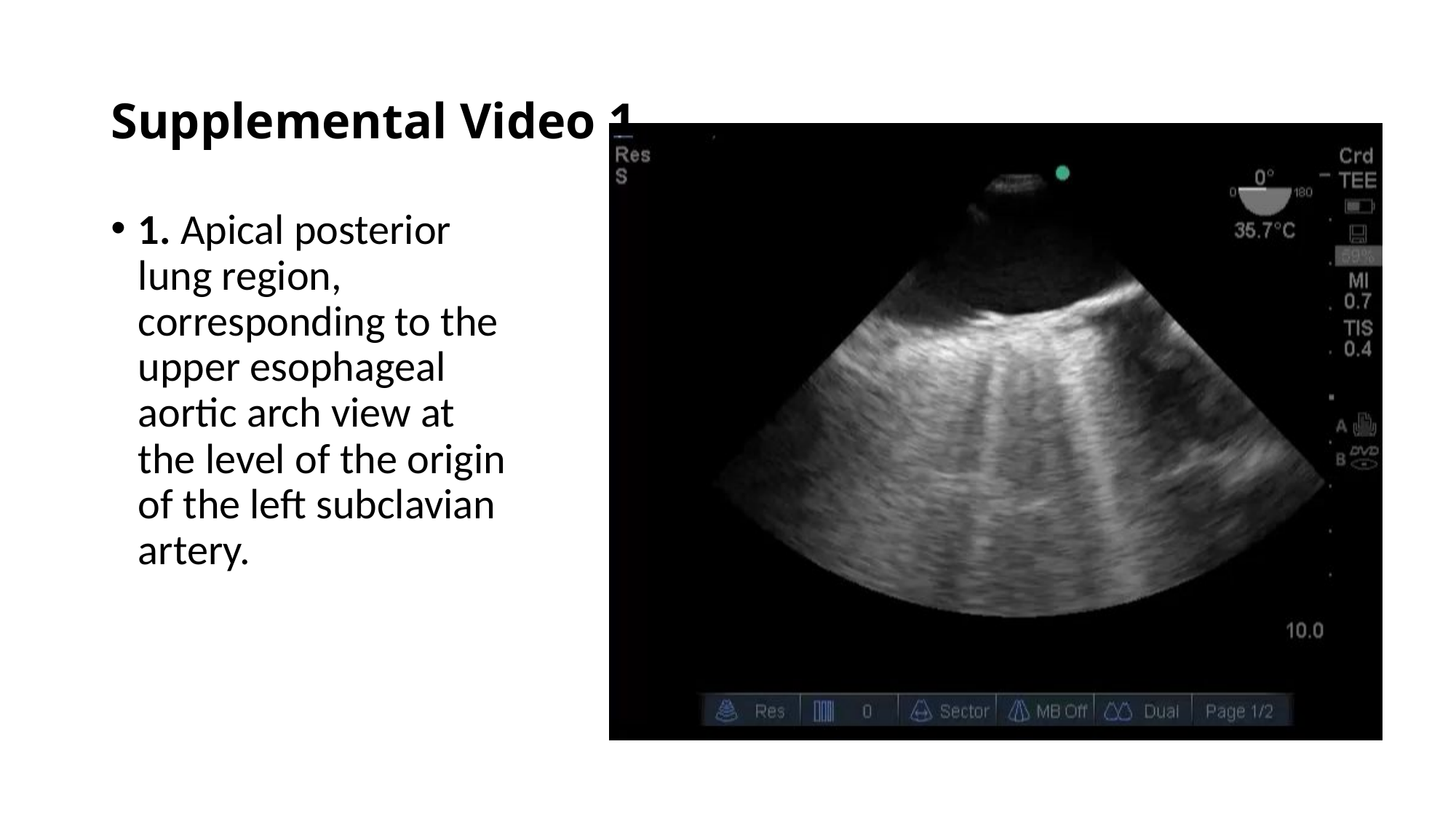

# Supplemental Video 1
1. Apical posterior lung region, corresponding to the upper esophageal aortic arch view at the level of the origin of the left subclavian artery.

## Slide 4
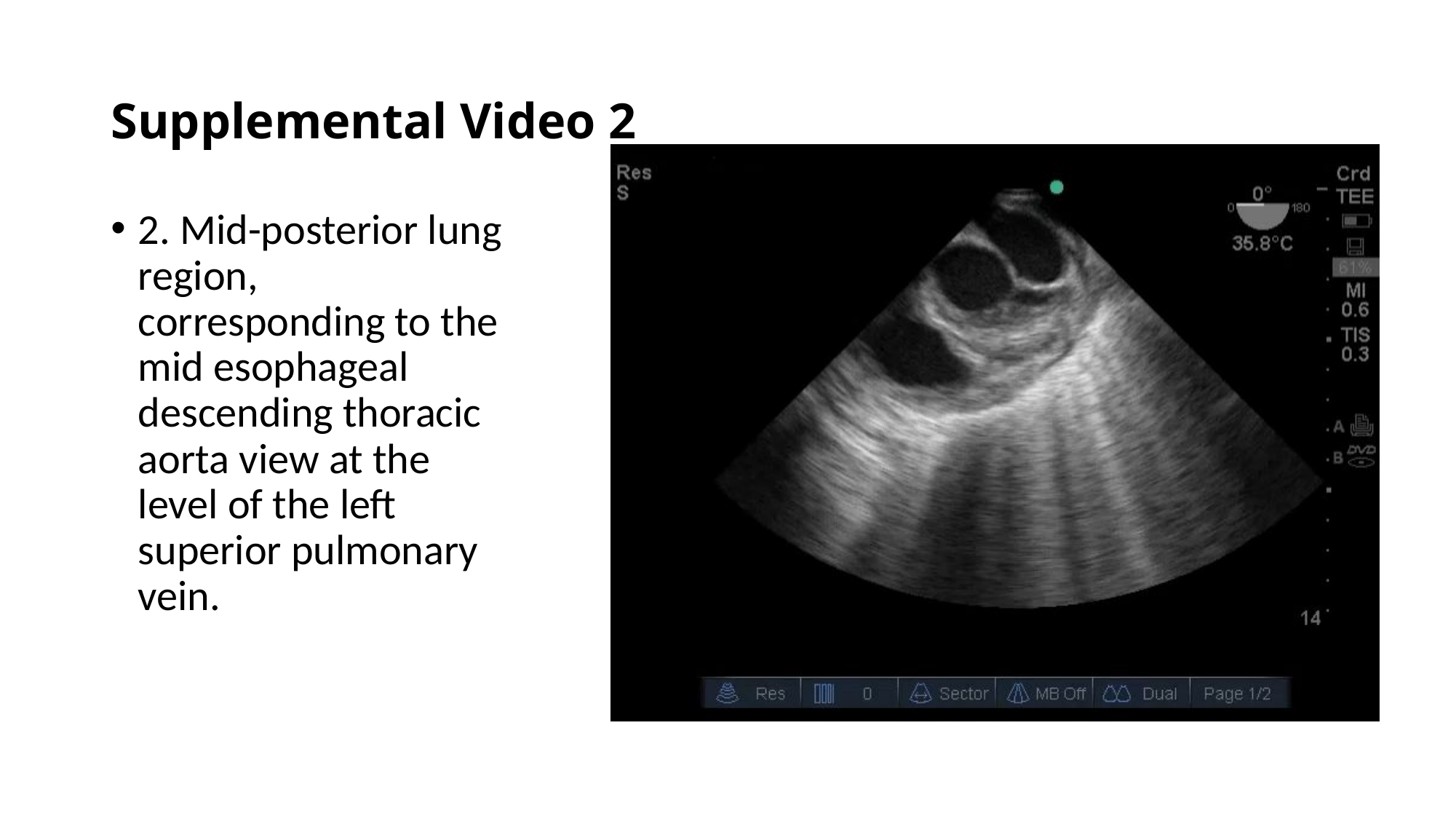

# Supplemental Video 2
2. Mid-posterior lung region, corresponding to the mid esophageal descending thoracic aorta view at the level of the left superior pulmonary vein.

## Slide 5
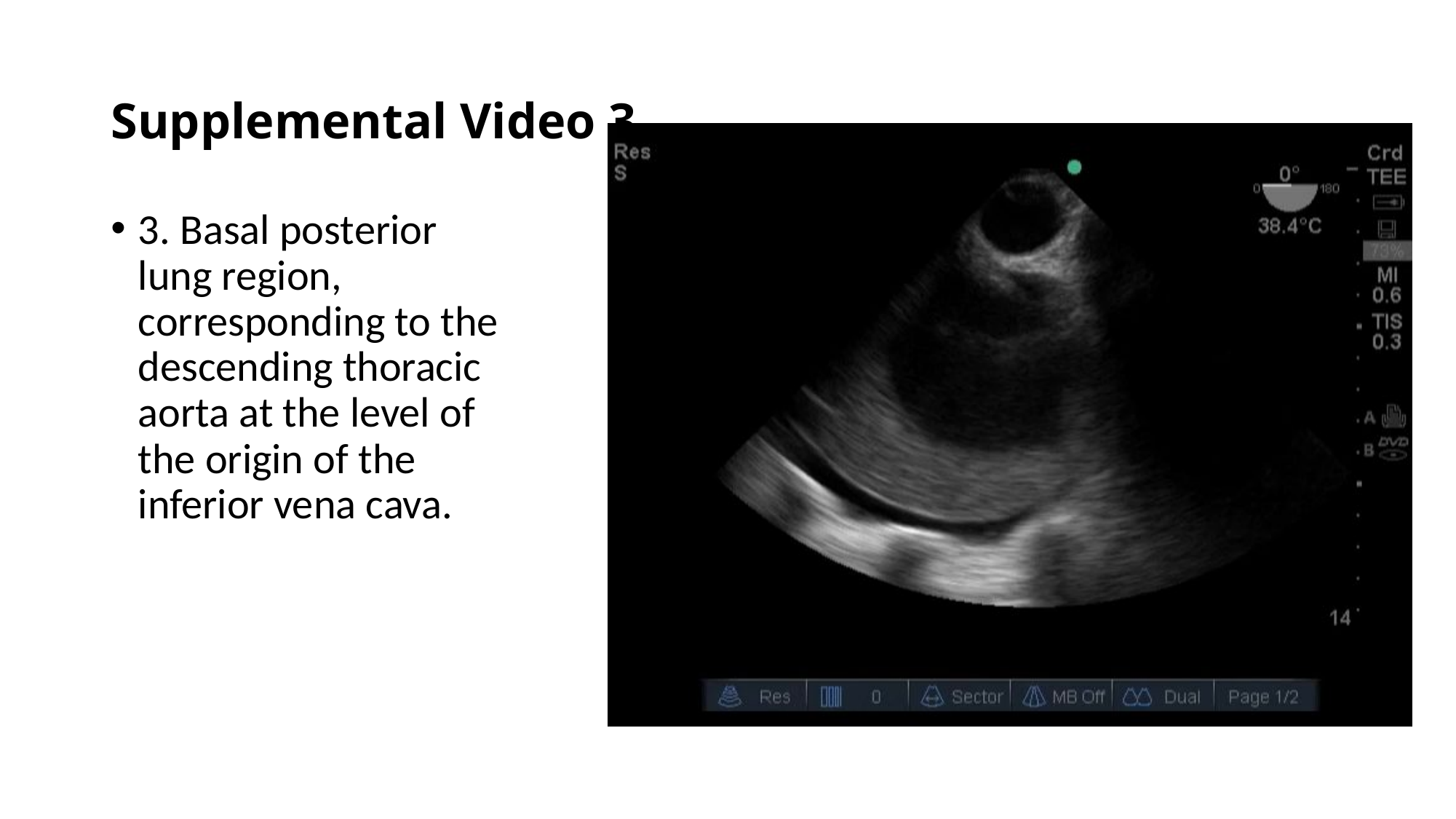

# Supplemental Video 3
3. Basal posterior lung region, corresponding to the descending thoracic aorta at the level of the origin of the inferior vena cava.
